# Risk of Malnutrition in Adults Who Have Undergone Sleeve Gastrectomy: A Retrospective Study

**DOI:** 10.3390/nu15173858

**Published:** 2023-09-04

**Authors:** Wan-Chun Liao, Tsae-Jyy Wang, Chieh-Yu Liu, Tsang-Pai Liu, Shu-Yuan Liang, Ko-Shih Chang

**Affiliations:** 1 Division of General, Department of Surgery, MacKay Memorial Hospital, Taipei City 10449, Taiwan; chunwanliao@gmail.com (W.-C.L.); liutp@mmh.org.tw (T.-P.L.); 2 School of Nursing, National Taipei University of Nursing and Health Sciences, Taipei City 11219, Taiwan; shuyuan@ntunhs.edu.tw; 3 Department of Health Care Management, National Taipei University of Nursing and Health Sciences, Taipei City 11219, Taiwan; chiehyu@ntunhs.edu.tw; 4 Division of Cardiovascular Medicine, Yuan Rung Hospital, Changhua 51045, Taiwan

**Keywords:** obesity, sleeve gastrectomy, nutritional status, prognostic nutritional index, hemoglobin, gastrointestinal symptoms, nutritional supplement

## Abstract

Sleeve gastrectomy achieves long-term weight control by reducing gastric volume. However, postoperative gastrointestinal symptoms and insufficient nutritional intake are likely to occur, which are not conducive to physical health. A retrospective study aimed to investigate changes in nutritional status and associated factors in patients after sleeve gastrectomy. Data were collected from the medical records of patients who underwent sleeve gastrectomy at a teaching hospital in Taiwan. Data from 120 patients who met the eligibility criteria were included in the analysis. The results show that sleeve gastrectomy has a strong weight loss effect. Within 12 months, the average body mass index of the patients decreased by 13.47 kg/m^2^. The number of morbidly obese patients decreased from 62 (51.7%) to 3 (2.5%). However, surgery is also associated with gastrointestinal symptoms and the threat of malnutrition. The number of patients with moderate to severe nutritional risk increased from 4 (3.3%) before surgery to 24 (20%) at 12-month follow-up. Likewise, the number of patients with anemia increased from 11 (9.2%) to 29 (24.17%). Gender, constipation, and diarrhea affected postoperative nutritional status. These findings suggest that patients after sleeve gastrectomy are at risk of malnutrition and require regular monitoring. Special attention should be given to women and patients with constipation or diarrhea, as they are at a particularly high risk of malnutrition.

## 1. Introduction

Obesity is a growing global health crisis. Statistics released by the World Health Organization (WHO) show that 39% of all adults are overweight [25 ≤ body mass index (BMI) < 29 kilogram/meter^2^ (kg/m^2^)] and 13% are obese (BMI ≥ 30 kg/m^2^) [1]. In Taiwan, the prevalence of being overweight and obese (BMI ≥ 27 kg/m^2^) is 50.6% and 23.9% [2]. Obesity is one of the causes or precipitating factors of many chronic illnesses, with roughly 70% of the world’s population dying prematurely from obesity-related diseases [3,4]. Weight control helps to prevent many complications of obesity-induced cardiovascular diseases and diabetes.

Surgical intervention for weight loss is recommended for patients with a BMI > 37.50 kg/m^2^ or with a BMI > 32.5 kg/m^2^ combined with hypertension, sleep apnea, or type 2 diabetes, if their glycosylated hemoglobin remains over 7.5% after medical treatment and lifestyle changes, or if a prescribed weight loss program fails to improve their situation within six months [5]. Weight loss procedures currently approved by the American Society for Metabolic and Bariatric Surgery include sleeve gastrectomy, biliopancreatic diversion with duodenal switch (BPD-DS), and Roux-en-Y gastric bypass (RYGB) [6]. Different procedures produce different weight loss effects and improve comorbidities to different degrees [7,8,9]. The risk of developing nutritional deficiencies also varies. Of the various bariatric surgery options that are clinically available, sleeve gastrectomy is one of the safest and most reliable [10]. In Taiwan, a total of 10,792 patients underwent sleeve gastrectomy in 2016–2021 [11].

Sleeve gastrectomy is the removal of approximately 66% of the stomach along the greater curvature, reducing the volume of the stomach to about 100 cm^3^ and altering its ability to absorb nutrients. The procedure also removes a large proportion of the cells in the stomach that produce the hunger hormone, ghrelin, thus reducing appetite and hunger [12,13]. These effects work in combination to help patients lose weight. Obese patients who undergo sleeve gastrectomy can expect to lose 59.47% of their body weight and 85% of their excess weight in about one year [10,14,15]. However, this procedure can also cause insufficient nutritional intake, resulting in protein, iron, folic acid, vitamin B12, and calcium deficiencies [16,17] and, in severe cases, anemia, muscle atrophy, and osteoporosis [18]. Monitoring changes in patients’ postoperative nutritional status is therefore crucial for adequate health education and care. 

A search for papers on nutritional changes in obese patients who had received sleeve gastrectomy published in 2018–2022 yielded two observational studies. One of these explored the protein intake of severely obese patients (*n* = 47) in the first three months after they underwent sleeve gastrectomy. It showed that the patients’ protein intake from food alone was significantly inadequate to meet their nutritional needs. The authors therefore concluded that protein supplementation was necessary after surgery to prevent nutritional deficiencies caused by insufficient protein intake [16]. The other study investigated anemia and related nutritional deficiencies in 82 patients 12 months after they had undergone sleeve gastrectomy. The results showed that the incidence of hypoalbuminemia decreased from 8.5% before surgery to 0% at 12 months post-surgery (*p* < 0.001). However, the incidence of anemia increased from 7.3% preoperatively to 11.0% at 12 months post-surgery (*p* = 0.109). This was accompanied by a significant increase in the prevalence of ferritin and serum iron deficiency. These findings suggest that postoperative anemia is a significant risk in sleeve gastrectomy [19]. 

Currently, there are no studies on the factors that affect patients’ nutritional status following sleeve gastrectomy, particularly the effects of postoperative gastrointestinal symptoms, nutritional consultation, and follow-up situation on postoperative nutritional status. In this study, we aimed to investigate changes in nutritional status and related factors in patients during the first year following sleeve gastrectomy. We used BMI, prognostic nutritional index (PNI), and hemoglobin as nutritional indicators, and the related factors we examined were age, gender, comorbidity status, hospital stay, number of bariatric outpatient visits, nutritional consultations, gastrointestinal symptoms, and nutritional supplement intake. 

## 2. Materials and Methods

### 2.1. Study Design

This study is a retrospective chart review. Research approval was granted by the institutional review board of the MacKay Memorial Hospital at which the study was conducted (approval no.: 22MMHIS299e). The need for informed consent was waived.

### 2.2. Samples and Location

The study included a consecutive sample of adults who underwent sleeve gastrectomy at the hospital, which is in Northern Taiwan, between 1 January 2016 and 31 December 2021. Patients who met the study eligibility criteria were selected based on data in the hospital’s electronic medical record (EMR) database and the admission and discharge logs of the general wards. The inclusion criteria were as follows: (1) aged between 20 and 55 years old, (2) diagnosed with obesity [International Classification of Diseases 10th Revision (ICD-10) E6601], and (3) at least one year since sleeve gastrectomy surgery. The exclusion criteria were as follows: (1) diagnosed with an adrenal or thyroid disorder, (2) history of substance abuse, and (3) incomplete nutritional profile in medical records. 

### 2.3. Data Collection and Instruments

The medical records of patients who underwent sleeve gastrectomy and filed claims for ICD-10 E6601 during the study period were retrieved from the hospital’s EMR and claims systems. Patients who met the inclusion criteria were selected and the necessary data were compiled into a datasheet, including the patients’ age, sex, comorbidity status, postoperative complications (anastomotic leakage, bleeding, and infection), length of hospitalization, number of bariatric outpatient visits in the first year after surgery, gastrointestinal symptoms (abdominal bloating, gastroesophageal reflux, nausea, vomiting, constipation, and diarrhea), and nutritional supplement intake. The patients’ nutritional status indicators were extracted from their test reports from before surgery (T0) and one month (T1), six months (T2), and 12 months (T3) post-surgery. The indicators extracted were hemoglobin, serum albumin, total lymphocyte count (TLC), and BMI.

The Charlson Comorbidity Index (CCI) [20] was used to assess comorbidity severity. The CCI encompasses 19 categories of medical conditions. The weighted index for each category is calculated based on the relative risk of death associated with that category, and the scores for each category are summed to derive the CCI. This is one of the most widely used methods of assessing comorbidity severity [20].

The PNI, calculated as 10 × serum albumin [grams per deciliter (g/dL)] + 0.005 × total lymphocyte count [cubic millimeter (mm^3^)], is a key indicator for evaluating patients’ nutritional status after gastrointestinal surgery. It also reflects their immune status, degree of systemic inflammation, and severity of postoperative complications. A PNI > 38 indicates no nutritional risk, 35 ≤ PNI ≤ 38 indicates moderate nutritional risk, and PNI < 35 indicates severe nutritional risk. Several studies conducted in other countries have found that TLC < 1000/mm^3^ combined with PNI < 35 is strongly correlated to poor nutritional status and a higher incidence of postoperative complications and mortality [21,22,23].

### 2.4. Data Analysis

SPSS Statistics 25.0 (IBM) was used for data entry and analysis. Central tendency and dispersion of the study variables were described using mean and standard deviation (SD), frequencies, and percentages. Generalized estimating equations (GEEs) were used to analyze changes in BMI, PNI, and hemoglobin over time and to examine the factors that affected postoperative BMI, PNI, and hemoglobin. The independent variables for the models were time, age, sex, comorbidity status, number of bariatric outpatient visits, number of nutritional consultations, gastrointestinal symptoms (abdominal bloating, gastroesophageal reflux, nausea, vomiting, constipation, and diarrhea), and nutritional supplement intake (multivitamins, iron, and protein powder).

## 3. Results

### 3.1. Patients’ Characteristics 

We retrieved data for 253 patients who had undergone sleeve gastrectomy during the study period. Of these, 133 were excluded because they were older than 55 years (*n* = 5), diagnosed with adrenal or thyroid disease (*n* = 10), had a history of substance abuse (*n* = 5), or did not have a complete nutritional profile in their medical records (*n* = 101). We therefore analyzed the data for 120 patients. Of these, 52 were male (43.3%) and 68 (56.7%) were female. Their mean age was 36.5 years (SD = 8.4). All patients were diagnosed as morbidly obese due to excess caloric intake. Of these, 56 also had sleep apnea, 73 had fatty liver, and 9 had polycystic ovary syndrome. Of these, 62 patients had cardiovascular disease and 46 had diabetes. The average CCI score was 1.6 (SD = 1.0), the average hospital stay was 6.6 days (SD = 0.9), and there were no postoperative complications. The mean number of bariatric clinic visits in the first postoperative year was 6.0 (SD = 1.4) and the average number of nutritional counseling sessions was 3.6 (SD = 0.8; Table 1).

### 3.2. Gastrointestinal Symptoms and Nutritional Supplement Intake

After surgery, the number of patients reporting all gastrointestinal symptoms that we investigated decreased over time, with the exception of diarrhea, which was reported by only one patient at T2 and T3 (Table 2). Bloating and gastroesophageal reflux are two of the most common gastrointestinal symptoms in patients. The number of patients taking nutritional supplements was low but increased overall with time after surgery (Table 2).

### 3.3. Postoperative Change in BMI and Its Key Predictors

**Figure 1:**
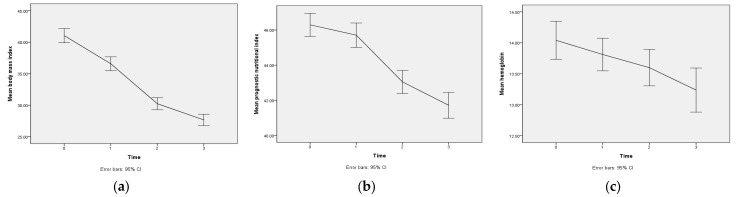
Line graphs of postoperative changes in (**a**) body mass index; (**b**) prognostic nutritional index; and (**c**) hemoglobin.

Our results indicated that the sleeve gastrectomy significantly reduced the body weight of the patients. The average BMI of the patients decreased significantly over time (Figure 1a). Compared with before surgery, the average BMI decreased by 4.55 kg/m^2^ 1 month after surgery, 10.89 kg/m2 at 6 months, and 13.47 kg/m^2^ at 12 months (Table 3). We categorized the BMI ranges into underweight (BMI < 18.5 kg/m^2^), healthy weight (18.5 ≤ BMI < 24 kg/m^2^), overweight (24 ≤ BMI < 27 kg/m^2^), pre-obese (27 ≤ BMI < 30 kg/m^2^), class I obesity (30 ≤ BMI < 35 kg/m^2^), class II obesity (35 kg/m^2^ ≤ BMI < 40 kg/m^2^), and class III obesity (BMI ≥ 40 kg/m^2^; Weir and Jan, 2022). Before surgery, 62 of the patients (51.7%) were in the class III obesity category. This figure decreased to 29 patients (24.2%) one month after surgery, 5 patients (4.2%) at six months, and 3 patients (2.5%) at 12 months. Chi-squared tests showed that these changes over time were statistically significant (*χ*^2^ = 294.4, *p* < 0.001; Table 2). The GEE analysis showed that the number of postoperative bariatric outpatient visits (*B* = − 0.2, *p* = 0.023) and diarrhea (*B* = 1.7, *p* = 0.001) were the key predictors of BMI. The effects of all of the other variables on BMI were not statistically significant (Table 4).

### 3.4. Postoperative Change in PNI and Its Key Predictors

We found that the sleeve gastrectomy increased the patients’ risk of malnutrition. The patients’ average PNI decreased over time (Figure 1b). Compared with before surgery, the average PNI decreased by 0.6 1 month after surgery, 3.2 at 6 months, and 4.6 at 12 months (Table 3). We categorized the PNI ranges into normal (>38), moderate (35 ≤ PNI ≤ 38), and severe (< 35) risk of malnutrition. Before surgery, only four patients (3.3%) were in the moderate and severe categories. This number increased to 8 patients (6.7%) 1 month after surgery, 12 patients (10%) at 6 months, and 24 patients (20%) at 12 months. Chi-squared tests showed that these changes over time were statistically significant (χ^2^ = 21.7, *p* = 0.001; Table 2). The GEE showed that constipation (B = 1.6, *p* = 0.036) and diarrhea (B = 3.0, *p* < 0.001) were the key predictors of PNI. The effects of all other variables on PNI were not statistically significant (Table 4).

### 3.5. Postoperative Change in Hemoglobin and Its Key Predictors

We found that the sleeve gastrectomy increased the risk of anemia. The patients’ average hemoglobin level decreased over time (Figure 1c). Compared with before surgery, the average hemoglobin level had decreased by 0.22 g/dL 1 month after surgery, 0.44 g/dL at 6 months, and 0.80 g/dL at 12 months (Table 3). Anemia was defined as hemoglobin level ≤13 g/dL in men and ≤12 g/dL in women. There were only 11 patients (9.2%) with anemia before surgery. This number increased to 13 patients (10.8%) 1 month after surgery, 19 patients (15.8%) at 6 months, and 29 patients (24.2%) at 12 months. Chi-squared tests showed that these differences were statistically significant (χ^2^ = 12.8, *p* = 0.005; Table 2). The GEE showed that sex (B = −1.7, *p* < 0.001), diarrhea (B = −1.49, *p* < 0.001), and iron supplementation (B = −1.8, *p* = 0.030) were the key predictors of hemoglobin levels. The effects of all other variables on hemoglobin were not statistically significant (Table 4).

## 4. Discussion

### 4.1. Postoperative Change in BMI and Its Key Predictors

We found that the sleeve gastrectomy significantly reduced both the obesity and the average BMI of the patients in this study. The degree of reduction was slightly lower than that reported by Omarov et al. [24], who examined patients (*n* = 57) in the Republic of Azerbaijan. They found that, on average, their patients’ BMI decreased by 7.4 kg/m^2^, 14.3 kg/m^2^, and 20.1 kg/m^2^ three, six, and twelve months after surgery compared to the average preoperative BMI. Notwithstanding the possibility of different eating habits, a potential reason for the discrepancy could be that the average preoperative BMI of the patients examined in this study (41.1 kg/m^2^) was lower than that reported in the 2020 study (46.7 ± 7.1 kg/m^2^).

We found that the key predictors of BMI were the number of postoperative bariatric outpatient visits and diarrhea. Patients with fewer outpatient visits were likely to have a smaller decrease in BMI. This supports the conclusion of Masood et al. [25], who asserted that patients who regularly receive postoperative outpatient follow-ups and whose nutritional status is monitored are better able to maintain their weight loss outcomes. Patients were also likely to exhibit a more rapid reduction in BMI if they had diarrhea. It may be that the diarrhea affected their ability to absorb nutrients, resulting in a more rapid decline in BMI. However, there is currently no relevant literature for comparison.

### 4.2. Postoperative Change in PNI and Its Key Predictors 

We found that the sleeve gastrectomy significantly reduced both the number of patients in the normal PNI range and the average PNI of the patients in this study. Constipation and diarrhea were the key predictors of PNI one year after surgery; patients with either of these symptoms were likely to have a poorer nutritional status than the other patients. There is currently no literature on the relationship between PNI and constipation or diarrhea. However, we infer that gastrointestinal symptoms such as these affect dietary intake, consequently increasing the risk of malnutrition.

### 4.3. Postoperative Change in Hemoglobin and Its Key Predictors 

We found that the sleeve gastrectomy significantly increased the number of patients who were anemic. This increase in anemia incidence was higher than that reported by Enani et al. [26], who analyzed 20 random controlled trial studies and found an incidence of iron deficiency anemia of 1.6% one year after sleeve gastrectomy. In contrast to this study, we evaluated anemia based on hemoglobin levels. The high proportion of patients with anemia that we observed can be attributed to reduced gastric acid secretion after surgery, hindering iron absorption and leading to iron deficiency anemia. In addition, factors such as underlying malnutrition before surgery (*n* = 4), diabetes combined with renal dialysis (*n* = 2), and reduced nutrient intake due to the restrictive surgery may lead to low hemoglobin levels.

The key predictors of hemoglobin levels were diarrhea, iron intake, and sex, such that patients were more likely to exhibit lower hemoglobin levels if they were female, had diarrhea, or took iron supplements. Notwithstanding the possibility of poorer nutrient intake and more severe obesity [27], the menstrual cycle can also affect hemoglobin levels in female patients. With respect to iron intake, further review of the patient records revealed that patients were prescribed iron supplements when their hemoglobin levels were 10 mg/dL or lower. It therefore seems likely that the iron supplementation was a consequence rather than a cause of low hemoglobin levels. A study in Australia reported an incidence rate of iron deficiency anemia caused by sleeve gastrectomy of 14–41.2%. Starting iron supplementation immediately after surgery can reduce the risk of anemia [28]. With respect to diarrhea, this symptom affects food and nutrient intake, consequently impacting hemoglobin levels. Further review of the patient records revealed that the patient with diarrhea took iron supplements in the same period. Therefore, we suspect that the diarrhea was caused by the intake of iron supplements for anemia. Sleeve gastrectomy reduces gastric acid secretion, hindering iron absorption and leading to iron deficiency anemia [29,30]. Therefore, regularly checking iron levels (including serum iron and total iron-binding capacity) is recommended for the diagnosis of iron deficiency anemia, early detection of potential risks, and provision of related nutritional interventions.

### 4.4. Gastrointestinal Symptoms and Nutritional Supplement Intake

Abdominal bloating was the most common gastrointestinal discomfort experienced by the patients, with 97.5%, 85.0%, and 30.0% of the patients reporting abdominal bloating one, six, and twelve months after surgery, respectively. These results support the conclusion of Wu et al. [18] that abdominal bloating is a prevalent postoperative gastrointestinal symptom in sleeve gastrectomy patients due to the reduction in stomach volume. Reflux was another common gastrointestinal symptom, with 80.8%, 71.7%, and 27.5% of the patients reporting gastroesophageal reflux one, six, and twelve months after surgery, respectively. These results support the conclusion of Felinska et al. [31] that patients are prone to developing gastroesophageal reflux within one year of undergoing sleeve gastrectomy because of the change in stomach capacity and the reduction in the baroreceptor reflex. Finally, 72.5%, 38.3%, and 5% of the patients reported nausea symptoms one, six, and twelve months after surgery, respectively. The incidence rates of all of these symptoms thus declined over time. These findings suggest that sleeve gastrectomy patients’ gastrointestinal status should be monitored and treated after surgery to improve their nutritional status.

Sleeve gastrectomy reduces stomach capacity and gastric secretions, which can affect patients’ ability to absorb vitamin B12, folic acid, iron, and other vitamins and minerals, leading to nutrient deficiencies. Patients can usually begin taking multivitamins two weeks after surgery. The Evidence-Based Guidelines for Adult Obesity Prevention and Control [31] recommend that patients receiving weight loss surgery take multivitamins in the long term to prevent nutrient deficiency. However, only 4.2%, 20.8%, and 29.2% of the patients in this study took multivitamin supplements one, six, and twelve months after surgery, respectively, highlighting the lack of education on nutritional supplementation after surgery. Sleeve gastrectomy removes approximately 66% of the stomach and causes a reduction in gastric acid secretion, hindering the patient’s ability to convert ferric iron in food to the more absorbable ferrous iron and increasing their risk of developing iron deficiency anemia. The Evidence-Based Guidelines for Adult Obesity Prevention and Control recommend that men without anemia and postmenopausal women take a multivitamin supplement daily after surgery to meet their nutritional requirements [32]. For women of childbearing age, the Guidelines recommend taking 45–60 mg of iron daily. Only a small proportion of the patients in this study took iron supplements after surgery (0.8% and 4.2% at T2 and T3). 

Because patients undergo intermittent fasting and experience rapid weight loss after weight loss surgery, they should begin supplementing with high-protein powder after returning home to prevent changes in their nutritional status caused by excessive protein consumption. Only a small proportion of the patients in this study took protein powder after surgery (1.7%, 4.2%, and 3.3% at T1, T2, and T3, respectively). This could be because some patients underwent intermittent fasting immediately after surgery and only began taking protein powder six months after surgery. Nine patients only began taking protein powder when their blood albumin dropped below 3.5 g/dL. Bertoni et al. [16] examined the protein intake of severely obese patients (*n* = 47) in the first three months after undergoing sleeve gastrectomy and found that they were receiving insufficient quantities of protein from the food that they consumed, resulting in nutritional deficiency. These researchers also found that increasing protein intake from food and supplementing with protein powder in the first month after surgery reduced the incidence of postoperative protein deficiency.

Further consideration should be given to whether there is an effect on intestinal structure. In addition, whether the malnutrition caused by gastrointestinal motility disorder in this population can be improved by simply taking medications, or whether it is necessary to improve the intestinal environment, should be looked at.

### 4.5. Study Limitations

There are several limitations to this study. First, we adopted a retrospective correlation research design and analyzed patient data from only one medical center. Therefore, the results of this study may not be representative of other patient groups. Second, we excluded 133 patients with incomplete nutritional profiles for at least one of the four analysis periods. This exclusion may have impacted our observations. Third, we only examined data concerning the patients’ gastrointestinal symptoms and nutritional supplementation. The lack of a comprehensive and systematic evaluation may have resulted in unexpected omissions. Fourth, serum iron levels were not evaluated during routine preoperative checkups. Therefore, we could not determine whether the patients had iron deficiency anemia prior to surgery. Fifth, although our results showed a significant effect of diarrhea on nutritional status, only one patient reported diarrhea, so this seems unlikely to be a significant predictor. Lastly, we tracked the changes in postoperative nutritional status for one year only. Extending the follow-up period could produce a clearer picture of the long-term postoperative nutritional and weight loss status of patients, particularly because postoperative weight gain is another potential risk associated with sleeve gastrectomy. Nonetheless, the findings provide important information on nutritional status and factors affecting it in adults undergoing sleeve gastrectomy.

## 5. Conclusions and Recommendations

Our findings support a strong weight control effect of sleeve gastrectomy. Within 12 months, the average BMI of the patients decreased by 13.47 kg/m^2^. The number of morbidly obese patients decreased from 62 (51.7%) to 3 (2.5%). However, this surgery is also associated with gastrointestinal symptoms and the threat of malnutrition. From preoperative to postoperative 12 months, the number of patients with moderate to severe nutritional risk increased from 4 (3.3%) to 24 (20%). Likewise, the number of patients with anemia increased from 11 (9.2%) to 29 (24.17%). These findings suggest the need for continued nutrition monitoring and support after sleeve gastrectomy patients are discharged from hospital. Female patients with constipation or diarrhea are at high risk of malnutrition. Clinicians and providers should pay particular attention to these high-risk populations. Strategies to maintain a balanced diet and interventions to relieve gastrointestinal symptoms are also recommended to improve the nutritional status of these postoperative patients. Particular attention should be paid to the intake of protein, iron, folic acid, and vitamin B12, as these postoperative patients are at risk of inadequate protein intake and anemia. 

## Figures and Tables

**Table 1:** Patients’ characteristics (*n* = 120).

Variables	*n*	%	
Sex			
Male	52	43.3	
Female	68	56.7	
Education level			
Middle school and below	10	8.3	
High school	37	30.8	
College and above	73	60.9	
Diagnosis			
Morbid obesity	120	100	
Sleep apnea	56	46.7	
Fatty liver	73	60.8	
Polycystic ovary syndrome	9	7.5	
	Mean	SD	Range
Age	36.5	8.4	20–55
Charlson Comorbidity Index	1.6	1.0	0–4
Number of outpatient visits	6.0	1.4	3–10
Number of nutritional consultations	3.6	0.8	3–6

SD, standard deviation.

**Table 2:** Gastrointestinal symptoms, nutritional supplement intakes, obesity, nutritional risk, and anemia in the first postoperative year (*n* = 120).

Variable	T0	T1	T2	T3	Chi-Square
	*n* (%)	*n* (%)	*n* (%)	*n* (%)	
Abdominal bloating	-	117 (97.5)	102 (85)	36 (30)	
Reflux symptoms	-	97 (80.8)	86 (71.7)	33 (27.5)	
Nausea	-	87 (72.5)	46 (38.3)	6 (5.0)	
Vomit	-	13 (10.8)	10 (8.3)	3 (2.5)	
Constipation	-	3 (2.5)	3 (2.5)	2 (1.7)	
Diarrhea	-	0	1 (0.8)	1 (0.8)	
Multivitamin intake	-	5 (4.2)	25 (20.8)	35 (29.2)	
Iron supplement intake	-	0 (0)	1 (0.8)	5 (4.2)	
Protein powder intake	-	2 (1.7)	5 (4.2)	4 (3.3)	
Level of obesity	-				294.4 ***
Underweight	0	0	0	1 (0.8)	
Healthy weight	0	0	13 (10.8)	23 (19.2)	
Overweight	0	3 (2.5)	18 (15.0)	35 (29.2)	
Pre-obese	0	13 (10.8)	28 (23.3)	33 (27.5)	
Obese class I	15 (12.5)	37 (30.8)	45 (37.5)	21 (17.5)	
Obese class II	43 (35.8)	38 (31.7)	11 (9.2)	4 (3.3)	
Obese class III	62 (51.7)	29 (24.2)	5 (4.2)	3 (2.5)	
Nutritional risk					21.7 **
Normal	116 (96.7)	112 (93.3)	108 (90)	96 (80)	
Moderate	3 (2.5)	6 (5.0)	11 (9.2)	20 (16.7)	
Severe	1 (0.8)	2 (1.7)	1 (0.8)	4 (3.3)	
Anemia status					12.8 *
Nonanemic	109 (90.8)	107 (89.2)	101 (84.2)	91 (75.8)	
Anemic	11 (9.2)	13 (10.8)	19 (15.8)	29 (24.2)	

T0, preoperative; T1, one month after surgery; T2, six months after surgery; T3, 12 months after surgery; -, not applicable; * *p* < 0.05; ** *p* < 0.01; *** *p* < 0.001.

**Table 3:** Patients’ body mass index, prognostic nutritional index, and hemoglobin changes over the first postoperative year (*n* = 120).

Variable	Time	Mean	SD	Range	GEE	
					B	95%CI
Body mass index	T3	27.7	5.0	18.0–49.6	−13.5 ***	−14.1~−12.9
	T2	30.2	5.3	21.10–53.5	−10.9 ***	−11.5~−10.3
	T1	36.6	6.1	25.0–57.6	−4.6 ***	−5.1~−4.0
	T0	41.0	6.2	32.4–65.7	0	
PNI	T3	41.7	4.1	30.1–50.2	−4.6 ***	−5.5~−3.7
	T2	43.1	3.6	32.1–52.2	−3.2 ***	−4.0~−2.5
	T1	45.7	3.8	30.2–53.1	−0.6 *	−1.1~−0.1
	T0	46.3	3.6	34.0–54.2	0	
Hemoglobin	T3	13.2	2.0	6.2–16.8	−0.8 ***	−1.1~−0.5
	T2	13.6	1.6	8.6–17.7	−0.4 ***	−0.7~−0.2
	T1	13.8	1.5	9.3–16.9	−0.2	−0.5~−0.0
	T0	14.0	1.7	9.0–17.4	0	

PNI, prognostic nutritional index; T0, preoperative; T1, one month after surgery; T2, six months after surgery; T3, 12 months after surgery; GEE, generalized estimating equation (model: (intercept), time; working correlation matrix structure: exchangeable); CI, Wald confidence interval; * *p* < 0.05; *** *p* < 0.001.

**Table 4:** Results of generalized estimating equations related to the predictors of body mass index, prognostic nutritional index, and hemoglobin in the first postoperative year (*n* = 120).

Independent Variable	Body Mass Index		Prognostic Nutritional Index		Hemoglobin	
Dependent Variable	B	SE	B	SE	B	SE
Intercept	44.1 ***	2.6	48.1 ***	1.3	14.7 ***	0.5
T3 vs. T0	−11.0 ***	0.9	−3.3 **	1.1	−1.2 ***	0.3
T2 vs. T0	−8.2 ***	0.9	−1.5	1.2	−0.8 **	0.3
T1 vs. T0	−1.7	0.9	1.4	1.1	−0.6	0.3
Age	−0.1	0.1	−0.0	0.0	0.0	0.0
Sex (female vs. male)	−0.6	1.0	−0.8	0.5	−1.7 ***	0.2
Charlson Comorbidity Index	1.0	0.6	0.2	0.3	0.1	0.1
Outpatient visits	−0.2 *	0.1	−0.1	0.1	0.0	0.0
Nutritional consultations	−0.3	0.2	−0.2	0.2	0.1	0.1
Bloating (yes vs. no)	−0.7	0.5	−0.5	0.5	−0.2	0.2
Reflux symptoms (yes vs. no)	0.1	0.3	−0.7	0.5	0.0	0.2
Nausea (yes vs. no)	0.1	0.4	−0.2	0.4	0.1	0.1
Vomit (yes vs. no)	0.4	0.5	−0.3	0.8	−0.2	0.3
Constipation (yes vs. no)	−0.3	0.6	1.6 *	0.8	−0.2	0.4
Diarrhea (yes vs. no)	1.7 **	0.5	3.0 ***	0.6	−1.5 ***	0.2
Multivitamin (yes vs. no)	0.5	0.5	0.7	0.6	−0.0	0.2
Iron supplement (yes vs. no)	1.7	0.9	−0.3	1.3	−1.8 *	0.8
Protein powder (yes vs. no)	1.3	1.4	1.1	0.7	0.1	0.3

Generalized estimating equation with working correlation matrix structure: exchangeable; T0, preoperative; T1, one month after surgery; T2, six months after surgery; T3, 12 months after surgery; * *p* < 0.05; **p < 0.01; *** *p* < 0.001.

## Data Availability

Data will be available from the corresponding author upon reasonable request.
